# Multiseptate gallbladder presenting with biliary colic

**DOI:** 10.1093/jscr/rjac417

**Published:** 2022-09-06

**Authors:** Peter Hopmann, Ellen Tan, David Lo

**Affiliations:** Department of General Surgery, OhioHealth Riverside Methodist Hospital, Columbus, OH, USA; Department of Internal Medicine, OhioHealth Riverside Methodist Hospital, Columbus, OH, USA; Department of Internal Medicine, OhioHealth Riverside Methodist Hospital, Columbus, OH, USA; Ohio Gastroenterology Group Inc., Columbus, OH, USA

## Abstract

Common diagnoses associated with right upper quadrant and epigastric pain include cholecystitis, peptic ulcer disease (PUD), biliary colic, gastroesophageal reflux disease (GERD) and irritable bowel syndrome (IBS). Multiseptate gallbladder is a rare congenital anomaly that can cause symptoms of biliary colic, however it may present with atypical symptoms, which can prolong definitive diagnosis and treatment. We present a case of multiseptate gallbladder in a 21-year-old female who initially presented with GERD and IBS. After multiple failed treatment regimens for IBS, she ultimately was found to have multiseptate gallbladder and was successfully treated with cholecystectomy.

## INTRODUCTION

Gallbladder disease is a frequent problem in the USA with a prevalence of 6% of men and 9% of women [1]. Gallbladder disease is most often related to development of gallstones with a variety of presentations including biliary colic, acute cholecystitis, choledocholithiasis, cholangitis and Mirizzi syndrome. The hallmark of gallbladder disease is right upper quadrant (RUQ) and/or epigastric pain. Definitive treatment of gallbladder disease is with cholecystectomy. Other common causes of RUQ and epigastric pain include peptic ulcer disease (PUD) and gastroesophageal reflux disease (GERD). Multiseptate gallbladder is a rare congenital disorder that may present with typical RUQ pain, but also may present with nonspecific symptoms, resulting in prolonged diagnosis and delay in definitive treatment. We present a case of multiseptated gallbladder that initially presented as GERD and was ultimately treated with cholecystectomy.

## CASE PRESENTATION

A 21-year-old female was referred to the gastroenterology clinic initially for evaluation of GERD and dysphagia. She noted 3 years of hoarseness and burning chest discomfort that had worsened over the past few months, along with intermittent solid food dysphagia. She had been placed on several proton pump inhibitors, but without complete resolution of her symptoms.

She was further evaluated with upper endoscopy only notable for erosive gastritis, and a duodenal biopsy that was negative for celiac disease. A few months later, she developed epigastric and RUQ abdominal pain and presented to the emergency department (ED) where a computed tomography (CT) scan showed signs of nonspecific colitis for which she was treated empirically with antibiotics. Subsequently, she was treated for IBS with trials of peppermint oil, and hyoscyamine along with multiple diet regimens including gluten, paleo and dairy-free over the next several months.

**Figure 1:**
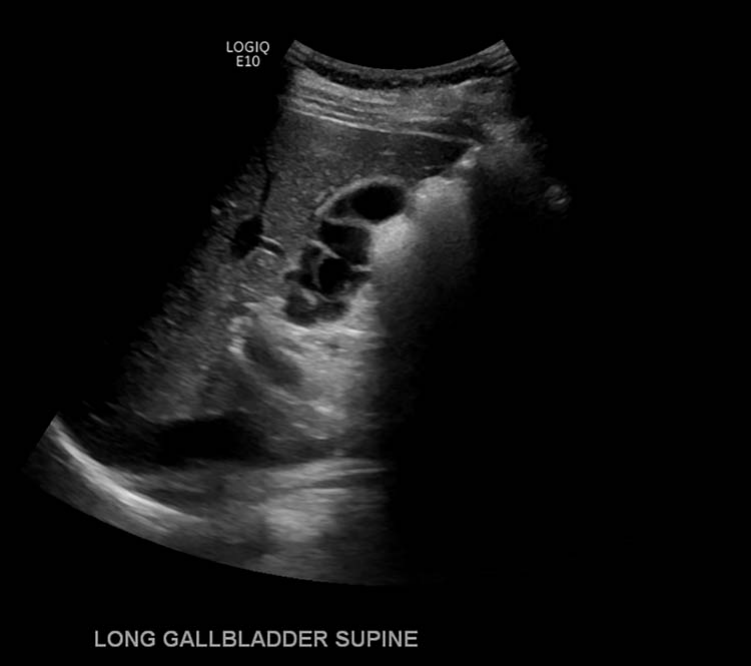
Ultrasound imaging of the gallbladder showing multiple septations resulting in a ‘sack of grapes’ appearance.

**Figure 2:**
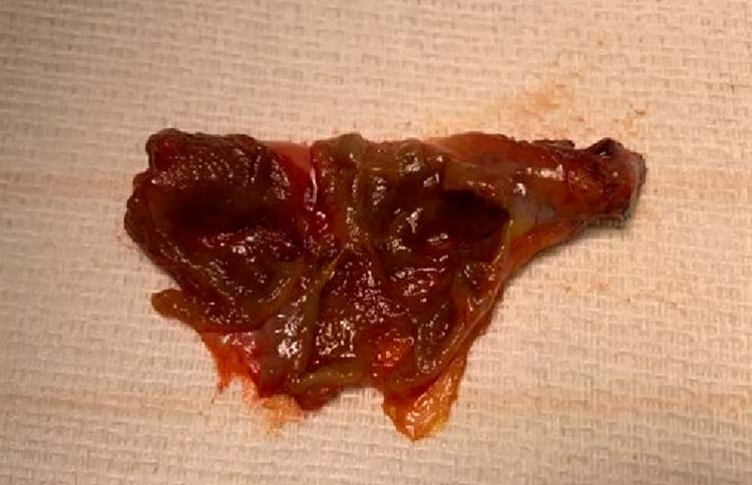
Gross specimen after cholecystectomy showing multiple internal septations and no stones.

Given continued abdominal pain, RUQ ultrasound was performed revealing a multiseptated gallbladder (Fig. 1). She uneventfully underwent a laparoscopic cholecystectomy, which revealed multiple septations on gross inspection consistent with multiseptate gallbladder (Fig. 2). Pathologic evaluation demonstrated changes of chronic cholecystitis. She was asymptomatic on postoperative follow-up.

## DISCUSSION

**Figure 3:**
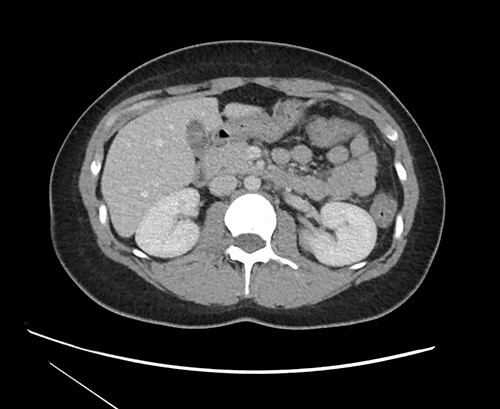
CT imaging at time of presentation to the ED showing multiple septations within the gallbladder.

Multiseptate gallbladder is a rare congenital anomaly, with only 57 published articles in the literature [2]. This entity was first identified in 1963 [3]. It may be asymptomatic, however the majority will develop symptoms of biliary colic at some point [4]. The septations can be visualized by multiple imaging modalities including ultrasound, magnetic resonance cholangiopancreatography (MRCP) and endoscopic retrograde cholangiopancreatography (ERCP) [4]. In the case of our patient, the multiple septa could also be visualized on CT imaging, though it was not described initially by the interpreting radiologist (see Fig. 3).

Patients with multiseptate gallbladder are more commonly symptomatic in adulthood, affecting females more than males [2, 5]. Patients may present with signs of nausea, vomiting and RUQ pain with the latter being the most common presenting complaint [2]. Liver function tests may also be elevated. Colic-like pain is thought to result from the incomplete emptying of the gallbladder due to the presence of multiple septations, or increased intraluminal pressure from dyssynchronous gallbladder contractions [2, 5, 6]. Multiple other pathologies of the biliary tree have been found to be associated with multiseptate gallbladder, including choledochal cysts (7% of reported cases), anomalous arrangement of the pancreaticobiliary duct, pancreatitis, cholelithiasis, gallbladder hypoplasia and even gallbladder cancer [2].

Multiple theories exist regarding pathophysiology of multiseptate gallbladder. The first is incomplete cavitation of the solid embryonic gallbladder resulting in multiple septations [2, 5]. However, this theory does not explain the presence of smooth muscle within the septal walls seen histologically in other cases [2, 5, 6]. The second theory is the Wrinkling theory, which suggests an irregular extrinsic wrinkling of the gallbladder wall, resulting in combining of the solid intraepithelial structures leading to septa formation [2, 5]. The third theory is the Phrygian cap theory, in which rapid growth of the gallbladder relative to its adjacent structures results in extrinsic compression from the peritoneal sac, ultimately leading to curling and flexing of the wall leading to fusion and septal formation [2, 5].

Regarding treatment, the standard of care is surgical cholecystectomy. In a review of 25 case reports, 17 out of the 25 patients experienced relief of their symptoms after cholecystectomy [7]. Similarly, our patient was treated with cholecystectomy and had resolution of her symptoms. This serves as a reminder to consider work-up of the biliary tree in patients with GERD and IBS symptomatology who fail medical management.
